# Serum MPO levels and activities are associated with angiographic coronary atherosclerotic plaque progression in type 2 diabetic patients

**DOI:** 10.1186/s12872-022-02953-7

**Published:** 2022-11-20

**Authors:** Qiujing Chen, Shuai Chen, Yang Dai, Xiaoqun Wang, Fenghua Ding, Ruiyan Zhang, Weifeng Shen, Wenbo Hu, Lin Lu, Wenqi Pan

**Affiliations:** 1 Department of Cardiovascular Medicine, Ruijin Hospital, Shanghai Jiao Tong University School of Medicine, 197 Ruijin Road II, Shanghai, 200025 China; 2 Institute of Cardiovascular Diseases, Shanghai Jiao Tong University School of Medicine, Shanghai, China; 3 Eachy Biopharma, Zhangjiagang, Jiangsu Province China

**Keywords:** MPO, Type 2 diabetes mellitus, Plaque progression

## Abstract

**Background:**

The uncontrolled production of MPO promotes inflammation, oxidative stress and atherosclerosis. Serum MPO levels are increased in patients with diabetes compared with patients without diabetes.

**Objectives:**

This study aimed to investigate whether the serum levels and activities of MPO are related to coronary plaque progression in patients with type 2 diabetes mellitus (T2DM).

**Material and methods:**

Serum MPO levels and activities were measured in 161 patients with diabetes with plaque progression (plaque progression group) and 87 patients with diabetes with no plaque progression (no plaque progression group). These patients were eligible based on the inclusion criteria and received quantitative coronary angiography at baseline and after approximately 1 year of follow-up. The characteristics and parameters of the participants at baseline were documented.

**Results:**

Serum MPO levels and activities were significantly higher in plaque progression group than in no plaque progression group (*P* < 0.001). We categorized these patients with diabetes into MPO level or activity tertile subgroups. Significant differences in the plaque progression ratio and prominent changes in the minimal lumen diameter, stenosis diameter and coronary artery stenosis score were observed across the tertile subgroups of MPO levels and activities (all *P* < 0.01). Moreover, serum MPO levels and activities correlated significantly with these indices of coronary artery disease severity after adjustment for other risk factors. Multivariable regression analyses revealed that serum MPO levels and activities remained independently associated with plaque progression, in addition to smoking, hypertension and CRP levels (all *P* < 0.05).

**Conclusions:**

Serum MPO levels and activities are significantly associated with coronary atherosclerotic plaque progression in patients with type 2 diabetes.

**Supplementary Information:**

The online version contains supplementary material available at 10.1186/s12872-022-02953-7.

## Introduction

It is well known that foam cells rich in cholesterol esters are characteristic of atherosclerotic plaques. High-density lipoprotein (HDL) particle-mediated cholesterol efflux is a crucial step in reverse cholesterol transport, antiatherogenesis and vascular protection [1]. Sufficient evidence has revealed that impaired HDL function by pathological modification of this lipoprotein or attenuated cholesterol transport contributes to atherosclerosis and plaque progression [1]. Atherosclerotic plaques progress to high-risk lesions, such as thin fibrous cap atherosclerosis, resulting in an increased risk of sudden death, ischemic stroke and acute myocardial infarction.

Patients with diabetes mellitus have a higher risk of vascular complications and cardiovascular events. In addition, once cardiovascular disease occurs, diabetes mellitus will aggravate the disease pathology and worsen the prognosis [2]. Importantly, the rate of atherosclerotic plaque development is faster in patients with diabetes mellitus [3]. Previous studies have suggested that metabolic disorders in the diabetic milieu cause overproduction of reactive oxygen species (ROS). ROS, via mechanisms of endothelial dysfunction and inflammation, play a major role in promoting diabetic vascular disease [4].

Myeloperoxidase (MPO) belongs to the heme-containing peroxidase family, which is mainly produced by polymorphonuclear neutrophils. Uncontrolled MPO release exacerbates inflammation, oxidative stress and metabolic disorders, leading to atherogenesis. Thus, the increased level or activity of MPO is a marker as well as a mediator involved in the pathophysiology of atherosclerosis [5]. Previous studies have demonstrated that MPO exerts a potential influence on atherosclerotic plaque rupture [6, 7]. MPO can enhance high glucose-induced vascular injury in diabetes [6, 7]. Importantly, MPO-mediated oxidation targets apolipoprotein A-I of HDL, which results in impaired reverse cholesterol transport of HDL [8]. In addition, serum MPO levels are higher in patients with diabetes than in patients without diabetes [9, 10]. These data have jointly suggested a hypothesis that MPO is closely related to atherosclerotic plaque progression in patients with diabetes.

Furthermore, most studies investigated only the total MPO levels, but rarely the MPO activity at the same time in coronary heart disease patients with T2DM. Thus, in the present study, to test the above hypothesis, we evaluated not only serum MPO levels but also MPO activity in T2DM patients with angiographically documented coronary plaque progression and in those without.

## Materials and methods

The research plan was approved by the ethics committee of Ruijin Hospital, Shanghai Jiao Tong University School of Medicine. All participants included in the study signed informed consent forms.

### Patients

**Fig. 1:**
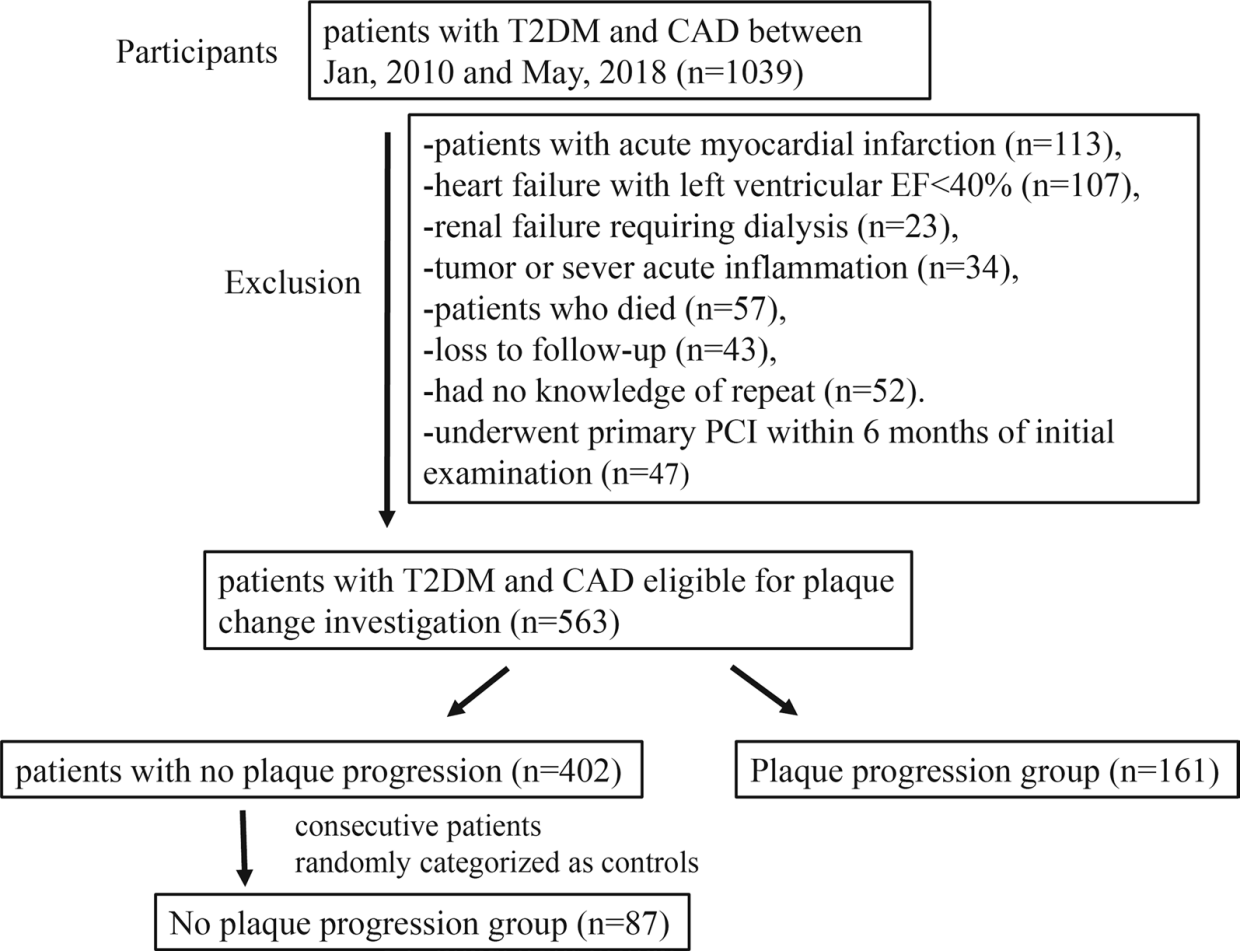
Flow chart of patient enrollment

The participants were patients with type 2 diabetes mellitus (T2DM) recruited from Shanghai Ruijin Hospital. From January 2010 to May 2018, a total of 1039 consecutive T2DM patients with coronary heart disease received PCI based on drug-eluting stents (Fig. 1). Coronary angiography and PCI for symptomatic patients with stable CAD was performed according to the indication of 2011 AHA guideline [11]. These diabetic patients with CAD received regular medications including statins and aspirin as suggested by guideline [11]. Patients with severe heart failure with left ventricular ejection fraction < 40% (n = 107), acute myocardial infarction (n = 113), renal failure requiring dialysis (n = 23), and tumor or severe acute inflammation (n = 34) were excluded from this study. Patients who were lost to follow-up (n = 43), died during follow-up (n = 57), and had no knowledge of repeat angiography (n = 52) were also excluded from the analysis. Another 47 patients who underwent primary PCI within six months of initial examination were also excluded from the study. The remaining 563 patients constituted the potential study population. These patients had at least one stenosis (lumen diameter stenosis > 20%) on the baseline coronary angiography that were located in the non-PCI interventional vessels and received follow-up angiography approximately one year later.

### Biochemical assessment

Blood samples were collected after fasting overnight. Serum was separated and stored at − 80 °C before use. Serum lipid levels (triglycerides [TGs], total cholesterol [TC], high density lipoprotein cholesterol [HDL-C], low density lipoprotein cholesterol [LDL-C], apolipoprotein A, apolipoprotein B and lipoprotein [Lp](a)) and glucose levels were measured by a HITACHI 912 automatic biochemistry analyzer (Roche Diagnostics, Germany). We used enzyme-linked immunosorbent assay (ELISA) (Biocheck Laboratory, USA) to detect the levels of serum C-reactive protein (CRP). MPO levels were determined by time-resolved fluorescence lateral flow immunoassay (TRFIA), which adopts the principle of immunochromatography and double antibody sandwich (Eachy biopharma, China). MPO activities were analyzed using a colorimetric assay kit (K744-100, BioVision, USA).

### Assessment of plaque progression

Coronary angiography and interventional therapy were performed by the standard Judkins technique or via the radial artery route [12]. Nitroglycerin (100 µg) was routinely injected into the coronary artery to avoid arterial spasm. In the initial and subsequent studies, coronary angiography recorded with the same projection was used for quantitative coronary artery analysis (QCA, Centricity Cardiology CA 1000. v1.0, USA). All images were analyzed by two well-experienced interventional specialists in heart diseases who knew nothing about the clinical data of the patients. The severity of coronary atherosclerosis was defined as single-vessel, double-vessel or triple-vessel disease according to the number of coronary vessels with severe stenosis (luminal stenosis > 50%).

For lesion analysis, end diastolic frames with the same angles were selected from the baseline and follow-up angiograms, which best showed the most severe stenosis, minimal anterior constriction, and branch overlap. The matching arterial segments were defined according to the images acquired from the anatomical positions of the proximal and distal branches. At follow-up, the segments with new lesions in non-PCI arteries and stenosis ≥ 20% at baseline, and all plaques with reference diameters ≥ 1.5 mm were analyzed. All coronary arteries involved in PCI were excluded to avoid including neointimal hyperplasia or restenosis after PCI. We measured the minimum lumen diameter (MLD) in diastole from multiple projections using the outer diameter of the contrast-filled catheter as a calibration. Finally, the results of a single worst view were recorded. The mean value was taken as the reference segment diameter from the 5 mm long, angiographically normal segment between the proximal and distal ends of the lesion but any major lateral branches. Atherosclerotic plaque progression was defined as: ≥ 1 lesion having a reduced MLD ≥ 0.4 mm (from baseline to follow-up angiography), which was approximately twice the SD of lesion repeated measurements [13]. The occurrence of a new coronary artery lesion was defined as no obvious stenosis on the initial angiography or stenosis diameter < 20%, but the reduction of MLD at follow-up angiography was ≥ 0.4 mm [14]. The coronary artery stenosis score (CCSS) was calculated based on the mean MLD of all measured segments in the coronary artery observed in each patient. Cumulative coronary artery occlusion was the sum of all diameter stenosis percentages expressed in standard index units (SI 50% = 0.50) [15]. Baseline QCA measurements minus follow-up measurements were defined as changes in QCA measurements.

Among 563 T2DM patients eligible for plaque progression analysis, 161 patients had plaque progression (plaque progression group). The other 402 patients had no plaque progression. Among these patients with no plaque progression, 87 consecutive patients were randomly referred to as the control (no plaque progression group).

### Statistical analyses

All statistical analyses were performed using SPSS for Windows 23.0 (SPSS Inc., Chicago, IL, USA). A 2-tailed *P* value < 0.05 was considered statistically significant. The Kolmogorov–Smirnov test was used for normal distribution evaluation. If the data were normally distributed, continuous variables were expressed as the mean ± standard deviation (SD); otherwise, they were expressed as the median (25th–75th percentile). Categorical variables are displayed as frequencies (percentages), and the differences between groups were compared by the chi-square test. When statistical calculation of normal distribution was needed, logarithmic transformation was performed for continuous variables of nonnormal distribution. When appropriate, unpaired t test, ANOVA, or nonparametric Mann–Whitney U test were used for intergroup comparisons. Pearson correlation was used to test the relationships between continuous variables, such as MPO level and activity and changes in QCA measurements. Spearman’s correlations were used to evaluate the relationships between continuous variables (such as MPO level and activity) and changes in MLD, stenosis diameter and CCSS. We used multivariable logistic regression models to estimate the independent risk factors for plaque progression.

## Results

### Clinical characteristics

All information for patients with plaque progression (plaque progression group) and those without plaque progression (no plaque progression group) are detailed in Table 1. In 563 patients with diabetes, coronary artery plaque progression occurred in 161 (28.6%). The plaque progression group had a significantly higher ratio of cigarette smoking and hypertension and increased CRP levels than the no progression group (all *P* < 0.01), but this was not observed for the duration and other parameters (Table 1). As expected, the changes in CCSS, MLD and stenosis diameter were significantly different between these two groups (all *P* < 0.001).

**Table 1:** Baseline characteristics and parameters in type 2 diabetic patients with plaque progression and those with no plaque progression

	No plaque progression (n = 87)	Plaque progression (n = 161)	*P* value
Male, n (%)	58 (66.7)	114 (70.8)	0.500
Age, years	65.71 ± 8.38	66.60 ± 9.77	0.476
Body mass index, kg/m^2^	24.63 ± 2.95	25.24 ± 3.31	0.154
Smoking, n (%)	17 (19.5)	65 (40.4)	0.001
Hypertension, n (%)	54 (62.1)	128 (79.5)	0.003
Dyslipidemia, n (%)	14 (16.1)	34 (21.1)	0.339
Severity of CHD, n (%)			0.217
1-Vessel	25 (28.7)	38 (23.6)	
2-Vessel	32 (36.8)	49 (30.4)	
3-Vessel	30 (34.5)	74 (46.0)	
Systolic blood pressure, mm Hg	138.74 ± 20.17	138.84 ± 19.85	0.967
Diastolic blood pressure, mm Hg	74.91 ± 16.37	75.42 ± 10.75	0.766
Fasting blood glucose, mmol/L	7.12 ± 1.80	7.04 ± 2.29	0.780
HbA1c, %	7.27 ± 1.17	7.43 ± 1.45	0.367
Serum creatinine, μmol/L	76.60 ± 19.79	84.14 ± 41.80	0.113
Serum BUN, mmol/L	6.96 ± 3.04	6.67 ± 3.53	0.509
Serum uric acid, μmol/L	334.90 ± 92.63	342.45 ± 91.04	0.536
eGFR, mL/min/1.73m^2^	84.64 ± 15.69	80.25 ± 18.56	0.062
Triglyceride, mmol/L	1.69 ± 0.88	1.60 ± 0.98	0.465
Total cholesterol, mmol/L	3.84 ± 1.17	3.96 ± 1.18	0.455
HDL cholesterol, mmol/L	1.08 ± 0.28	1.06 ± 0.27	0.581
LDL cholesterol, mmol/L	2.22 ± 0.89	2.37 ± 0.95	0.216
Apolipoprotein A, g/L	1.25 ± 0.23	1.20 ± 0.23	0.095
Apolipoprotein B, g/L	0.74 ± 0.22	0.79 ± 0.23	0.109
Lipoprotein (a), g/L	0.22 ± 0.24	0.25 ± 0.26	0.458
CRP, mg/mL	0.63 (0.28–2.43)	1.14 (0.49–4.08)	0.001
Medication, n (%)			
ACE inhibitors/ARBs	55 (63.2)	112 (69.6)	0.309
β-blockers	43 (49.4)	96 (59.6)	0.122
Statins	80 (92.0)	146 (90.7)	0.985
Antiplatelet	82 (94.3)	154 (95.7)	0.758
Metformin	31 (35.6)	54 (33.5)	0.740
Insulin	19 (21.8)	36 (22.4)	0.925
MPO level, ng/mL	20.20 (19.20–27.00)	33.30 (21.45–60.00)	< 0.001
MPO activity, mU/mL	6.94 ± 5.20	10.37 ± 7.26	< 0.001
Follow-up			
Duration, months	12.18 ± 1.29	12.32 ± 1.24	0.428
Changes in MLD, mm	− 0.06 ± 0.09	0.67 ± 0.25	< 0.001
Changes in stenosis diameter (%)	0.88 ± 3.29	− 22.70 ± 9.86	< 0.001
Changes in CCSS	− 0.01 ± 0.08	− 0.33 ± 0.15	< 0.001

Values are given as mean ± standard deviation (SD), median (25th–75th percentile) or number (percentage)

Change of QCA measurement is defined as baseline QCA measurement minus follow-up measurement

*CHD* Coronary atherosclerotic heart disease, *ACE* Angiotensin converting enzyme, *ARB* Angiotensin receptor blocker, *BUN* Blood urea nitrogen, *CRP* C-reactive protein, *eGFR* Estimated glomerular filtration rate, *HbA1c* Glycosylated hemoglobin, *HDL* High-density lipoprotein, *LDL* Low-density lipoprotein, *CCSS* Cumulative coronary stenosis score, *MLD* Minimal lumen diameter, *QCA* Quantitative coronary analyses

### MPO levels and activities

The plaque progression group had significantly higher MPO levels than the no plaque progression group [33.30 (21.45–60.00) ng/mL vs. 20.20 (19.20–27.00) ng/mL, *P* < 0.001]. MPO activities in the plaque progression group were also significantly higher than those in the no plaque progression group [10.37 ± 7.26 mU/mL vs. 6.94 ± 5.20 mU/mL, *P* < 0.001]. We further categorized the patients into tertile subgroups according to MPO levels and activities (Tables 2, 3). Baseline data of tertile subgroups are provided in Additional file 1: Table S1 and Additional file 2: Table S2. Notably, significant differences in plaque progression ratio, change in MLD, diameter stenosis and CCSS were observed across the tertile subgroups of MPO levels and activities (all *P* < 0.01) (Tables 2, 3). Moreover, serum MPO levels and activities correlated significantly with changes in MLD, diameter stenosis and CCSS before and after adjustment for parameters including age, sex, body mass index (BMI), history of hypertension, smoking, estimated glomerular filtration rate (eGFR), HDL cholesterol (HDL-C), LDL cholesterol (LDL-C), Log CRP levels, antiplatelet and statin use (all *P* < 0.001) (Table 4). MPO levels and activities for 1-vessel vs. 2-vessel vs. 3-vessel disease in patients with CHD (coronary atherosclerotic heart disease) are shown in Additional file 3: Table S3. There was a significant difference in MPO activity among the three groups (*P* < 0.05).

**Table 2:** Plaque progression in different tertiles of MPO levels in patients with type 2 diabetes

Tertiles of MPO levels	n	Follow-up duration, (months)	Plaque progression, n (%)	Changes in MLD, (mm)	Changes in stenosis diameter, (%)	Changes in CCSS
T1	84	12.18 ± 1.44	34 (42.0)	0.17 ± 0.26	− 4.37 ± 6.82	− 0.11 ± 0.11
T2	82	12.26 ± 1.26	54 (65.9)	0.34 ± 0.32	− 13.52 ± 12.97	− 0.18 ± 0.15
T3	82	12.38 ± 1.05	73 (85.9)	0.74 ± 0.40	− 25.44 ± 12.14	− 0.38 ± 0.22
*P* value		0.591	< 0.001	< 0.001	< 0.001	< 0.001

Values are given as mean ± standard deviation (SD) or number (percentage)

Change of QCA measurement is defined as baseline QCA measurement minus follow-up measurement

Tertiles of MPO levels: T1 ≤ 20.500 ng/mL; 20.500 < T2 ≤ 38.317 ng/ mL; T3 > 38.317 ng/mL

*MLD* Minimal lumen diameter, *CCSS* Cumulative coronary stenosis score, *QCA* Quantitative coronary analyses

**Table 3:** Plaque progression in different tertiles of MPO activity in patients with type 2 diabetes

Tertiles of MPO activity	n	Follow-up duration, (months)	Plaque progression, n (%)	Changes in MLD, (mm)	Changes in stenosis diameter, (%)	Changes in CCSS
T1	83	12.22 ± 1.08	43 (51.8)	0.28 ± 0.40	− 9.08 ± 13.23	− 0.14 ± 0.19
T2	82	12.16 ± 0.84	52 (63.4)	0.40 ± 0.39	− 13.69 ± 13.57	− 0.21 ± 0.19
T3	83	12.43 ± 1.69	66 (79.5)	0.58 ± 0.37	− 20.51 ± 12.65	− 0.31 ± 0.20
*P* value		0.335	0.001	< 0.001	< 0.001	< 0.001

Values are given as mean ± standard deviation (SD) or number (percentage)

Change of QCA measurement is defined as baseline QCA measurement minus follow-up measurement

Tertiles of MPO activity: T1 ≤ 6.0307 mU/mL; 6.0307 < T2 ≤ 9.8684 mU/mL; T3 > 9.8684 mU/mL

*MLD* Minimal lumen diameter, *CCSS* Cumulative coronary stenosis score, *QCA* Quantitative coronary analyses

**Table 4:** Correlation of serum levels and activity of MPO with plaque progression in patients with type 2 diabetes

	Changes in MLD	Changes in stenosis diameter	Changes in CCSS
Serum levels of MPO, ng/mL			
Unadjusted r	0.62	− 0.62	− 0.59
Unadjusted P	< 0.001	< 0.001	< 0.001
Adjusted r*	0.53	− 0.44	− 0.56
Adjusted P*	< 0.001	< 0.001	< 0.001
Serum MPO activity, mU/mL			
Unadjusted r	0.38	− 0.37	− 0.38
Unadjusted P	< 0.001	< 0.001	< 0.001
Adjusted r*	0.30	− 0.31	− 0.33
Adjusted P*	< 0.001	< 0.001	< 0.001

Change of QCA measurement is defined as baseline QCA measurement minus follow-up measurement

*CRP* C-reactive protein, *HDL* High-density lipoprotein, *LDL* Low-density lipoprotein, *CCSS* Cumulative coronary stenosis score, *MLD* Minimal lumen diameter, *QCA* Quantitative coronary analyses

*Adjusted for gender, age, body mass index, history of hypertension, smoking, glycated hemoglobin A1c, estimated glomerular filtration rate, HDL cholesterol, LDL cholesterol, Log CRP, antiplatelet and statins use

### Multivariable logistic regression analysis

Multivariable logistic regression analysis was performed to ascertain the risk factors for plaque progression in the patients with diabetes by including all the factors in Table 1. The results revealed that smoking, hypertension and CRP were independent determinants of plaque progression (Model 1). After inclusion of MPO levels and activities and adjustment for these variables (Models 2 and 3), MPO levels and activities remained independently associated with plaque progression (MPO levels in Model 2, OR for T2: 2.10, 95% CI 1.04–4.24, *P* < 0.05; OR for T3: 7.41, 95% CI 3.29–16.71, *P* < 0.001) (MPO activities in Model 3, OR for T2: 1.78, 95% CI 0.89–3.57, *P* = 0.101; OR for T3: 3.53, 95% CI 1.67–7.47, *P* < 0.01) (Table 5). Compared with Model 1, the addition of MPO levels significantly improved the C statistic by 0.06 in Model 2 [from 0.73 (95% CI 0.66–0.79) to 0.79 (95% CI 0.73–0.85)] and MPO activities by 0.02 in Model 3 [from 0.73 (95% CI 0.66–0.79) to 0.75 (95% CI 0.69–0.81)].

**Table 5:** Multivariable logistic regression analysis for the risk of plaque progression in patients with type 2 diabetes

Variables	Adjusted OR (95%CI)	*P* value
Model 1		
Male	1.08 (0.55–2.12)	0.835
Age	1.00 (0.96–1.03)	0.884
Body mass index	1.07 (0.98–1.17)	0.158
Smoking	2.48 (1.25–4.90)	0.009
Hypertension	2.03 (1.09–3.79)	0.026
HbA1c, %	1.09 (0.87–1.36)	0.464
eGFR, mL/min/1.73m^2^	0.98 (0.96–1.00)	0.069
HDL cholesterol, mmol/L	0.75 (0.25–2.24)	0.611
LDL cholesterol, mmol/L	1.16 (0.82–1.62)	0.403
Log-transferred CRP	2.02 (1.21–3.36)	0.007
Antiplatelet	0.78 (0.21–2.94)	0.713
Statins	0.77 (0.27–2.18)	0.618
Model 2		
Male	0.88 (0.42–1.85)	0.738
Age	1.00 (0.97–1.04)	0.930
Body mass index	1.05 (0.95–1.15)	0.376
Smoking	2.64 (1.28–5.42)	0.008
Hypertension	2.21 (1.13–4.32)	0.020
HbA1c, %	1.06 (0.84–1.34)	0.623
eGFR, mL/min/1.73m^2^	0.98 (0.96–1.00)	0.111
HDL cholesterol, mmol/L	0.78 (0.24–2.54)	0.684
LDL cholesterol, mmol/L	1.18 (0.82–1.70)	0.371
Log-transferred CRP	1.72 (1.00–2.94)	0.049
Antiplatelet	0.64 (0.17–2.43)	0.514
Statins	0.83 (0.27–2.51)	0.739
Tertiles of MPO level		< 0.001
T1	1 (reference)	/
T2	2.10 (1.04–4.24)	0.038
T3	7.41 (3.29–16.71)	< 0.001
Model 3		
Male	1.13 (0.57–2.27)	0.722
Age	1.00 (0.96–1.04)	0.890
Body mass index	1.05 (0.96–1.15)	0.299
Smoking	2.34 (1.16–4.71)	0.017
Hypertension	1.99 (1.05–3.79)	0.036
HbA1c, %	1.07 (0.85–1.34)	0.564
eGFR, mL/min/1.73m^2^	0.98 (0.96–1.00)	0.047
HDL cholesterol, mmol/L	0.74 (0.24–2.29)	0.606
LDL cholesterol, mmol/L	1.21 (0.86–1.72)	0.277
Log-transferred CRP	1.97 (1.16–3.32)	0.012
Antiplatelet	0.62 (0.16–2.42)	0.488
Statins	0.67 (0.23–1.97)	0.470
Tertiles of MPO activity		0.004
T1	1 (reference)	/
T2	1.78 (0.89–3.57)	0.101
T3	3.53 (1.67–7.47)	0.001

Tertiles of MPO levels: T1 ≤ 20.500 ng/mL; 20.500 < T2 ≤ 38.317 ng/mL; T3 > 38.317 ng/mL

Tertiles of MPO activity: T1 ≤ 6.0307 mU/mL; 6.0307 < T2 ≤ 9.8684 mU/mL; T3 > 9.8684 mU/mL

*CRP* C-reactive protein, *eGFR* Estimated glomerular filtration rate, *HbA1c* Glycosylated hemoglobin, *HDL* High-density lipoprotein, *LDL* Low-density lipoprotein

## Discussion

The uncontrolled release of MPO promotes inflammation, oxidative stress and cardiovascular diseases. Our study demonstrated that increased MPO levels and activities are associated with plaque progression. Serum MPO levels and activities were correlated with changes in MLD, stenosis diameter and CCSS before and after adjustment or conventional risk factors. Multivariable logistic regression analysis revealed that serum MPO levels and activities are independent determinants of plaque progression in patients with diabetes.

MPO is a heme-containing enzyme produced in neutrophils. It catalyzes reactions of which products and their secondary metabolites are responsible for killing bacteria and viruses. However, uncontrolled MPO release exaggerates inflammation, oxidative stress and metabolic disorders, leading to cardiovascular diseases and tissue damage [5]. Previous studies have demonstrated that MPO exerts a potential influence on many cardiovascular diseases, including atherosclerotic plaque rupture, diabetic vasculopathy, myocardial ischemia/reperfusion injury, hypertension, heart failure, pulmonary arterial hypertension, stroke, cardiac arrhythmia, venous thrombosis [6, 7, 16], and chronic kidney disease [17]. Some studies have suggested that elevated MPO levels predict future risk of coronary artery disease in apparently healthy individuals, but some do not [18, 19]. Serum MPO level is closely associated with the biological activity of MPO [20]. Thus, MPO is a promoter, mediator and marker of cardiovascular diseases.

Moreover, serum levels of MPO are elevated in patients with diabetes compared with patients without diabetes [9]. Fasting plasma glucose levels are positively correlated with plasma MPO levels [21]. MPO strongly oxidizes apolipoprotein A-I of HDL in patients with diabetes, which results in rampant impairment of cholesterol transport [8, 9]. A previous study has showed that HDL function is significantly altered in patients with diabetes with peripheral atherosclerosis disease due to multiple modifications of this lipoprotein that are aggravated by diabetes. Thus, plasma glucose and MPO levels constitute indicators of HDL dysfunction and contribute to risk stratification in patients with diabetes [22].

In the present study, we found for the first time that serum MPO activities were significantly associated with angiographically documented coronary artery plaque progression in patients with diabetes. The serum MPO levels were consistent with those of a previous study [23] and the abovementioned findings. Thus, these data suggest that MPO is closely associated with atherosclerotic plaque progression in patients with diabetes, which is significant in the prognostic implication in diabetes.

### Limitations

We acknowledge that there are limitations in this study. First, our study is a cross-sectional study, aiming to investigate the relationship between MPO and coronary atherosclerotic plaque progression but not causative links. Second, FMD-closely associated apoA-I has various modification forms in patients with diabetes, such as glycation. Thus, impairment of cholesterol transport could be a combined effect of different apoA-I modifications, which jointly exert influence on plaque development. MPO-mediated oxidation is only one aspect of influence on plaques. In our future studies, a prospective study regarding the relationships between atherosclerotic plaque progression and substrates of various apoA-I modifications, including MPO, will be investigated.

## Conclusion

Serum MPO levels and activities are associated with coronary atherosclerotic plaque progression in T2DM patients, suggesting that MPO is a crucial mediator or marker of atherosclerotic plaque development.

## Supplementary Information

**Additional file 1. Table S1.** Baseline characterize of patients with type 2 diabetes in different tertiles of MPO levels.

**Additional file 2. Table S2.** Baseline characterize of patients with type 2 diabetes in different tertiles of MPO activity.

**Additional file 3. Table S3.** MPO levels and activities for 1-vessel versus 2-vessel versus 3-vessel disease in patients with CHD.

## Data Availability

The datasets analyzed in this study are available from the corresponding authors upon reasonable request.
