# Supplementary material for: Serum MPO levels and activities are associated with angiographic coronary atherosclerotic plaque progression in type 2 diabetic patients
Source: BMC Cardiovasc Disord. 2022 Nov 20;22:496. doi: 10.1186/s12872-022-02953-7 (PMC9677674; doi:10.1186/s12872-022-02953-7)
Supplement: Supplementary file 1 — Additional file 1. Table S1. Baseline characterize of patients with type 2 diabetes in different tertiles of MPO levels. [file 12872_2022_2953_MOESM1_ESM.docx]

**Table S1.** **Baseline characterize of patients with type 2 diabetes in different tertiles of MPO levels.**

|  | Diabetic patients | | | |
| --- | --- | --- | --- | --- |
| Tertiles of MPO levels | T1(n=84) | T2(n=82) | T3(n=82) | P value |
| male, n (%) | 51(60.7) | 60(73.2) | 61(74.4) | 0.106 |
| age, years | 67.14±9.37 | 65.89±8.31 | 65.80±10.16 | 0.584 |
| body mass index, kg/m^2^ | 24.73±3.16 | 24.83±3.16 | 25.53±3.25 | 0.220 |
| smoking, n (%) | 19(22.6) | 36(43.9) | 27(32.9) | 0.014 |
| hypertension, n (%) | 58(69.0) | 64(78.0) | 60(73.2) | 0.422 |
| dyslipidemia, (%) | 14(16.7) | 15(18.3) | 19(23.2) | 0.545 |
| systolic blood pressure, mm Hg | 139.90±20.60 | 138.30±19.66 | 138.18±19.67 | 0.825 |
| diastolic blood pressure, mm Hg | 75.32±16.43 | 74.83±11.40 | 75.57±10.31 | 0.933 |
| fasting blood glucose, mmol/L | 7.07±1.85 | 7.05±2.21 | 7.07±2.32 | 0.998 |
| HbA1c, % | 7.33±1.23 | 7.27±1.35 | 7.52±1.50 | 0.484 |
| serum creatinine, μmol/L | 76.23±17.85 | 87.57±54.15 | 80.82±24.11 | 0.121 |
| serum BUN，mmol/L | 6.86±3.03 | 7.05±3.86 | 6.41±3.15 | 0.455 |
| serum uric acid, μmol/L | 343.83±95.59 | 324.93±91.40 | 350.54±86.31 | 0.178 |
| eGFR, mL/min/1.73m^2^ | 82.53±16.14 | 80.62±18.95 | 82.21±18.08 | 0.761 |
| triglyceride, mmol/L | 1.87±1.23 | 1.42±0.62 | 1.58±0.84 | 0.007 |
| total cholesterol, mmol/L | 3.90±1.21 | 3.96±1.14 | 3.88±1.19 | 0.915 |
| HDL cholesterol, mmol/L | 1.07±0.30 | 1.10±0.26 | 1.02±0.25 | 0.146 |
| LDL cholesterol, mmol/L | 2.30±1.00 | 2.32±0.86 | 2.34±0.94 | 0.978 |
| apolipoprotein A, g/L | 1.24±0.25 | 1.24±0.21 | 1.18±0.22 | 0.132 |
| apolipoprotein B, g/L | 0.76±0.24 | 0.78±0.23 | 0.77±0.22 | 0.832 |
| lipoprotein (a), g/L | 0.23±0.24 | 0.26±0.23 | 0.23±0.28 | 0.671 |
| CRP, mg/mL | 0.63(0.34-1.82) | 1.19(0.44-3.96) | 1.33(0.50-3.76) | 0.006 |
| medication, n (%) |  |  |  |  |
| ACE inhibitors/ARBs | 52(61.9) | 56(68.3) | 59(72.0) | 0.376 |
| β-blockers | 43(51.2) | 48(58.5) | 48(58.5) | 0.544 |
| statins | 76(90.5) | 77(93.9) | 73(89.0) | 0.529 |
| antiplatelet | 77(91.7) | 79(96.3) | 80(97.6) | 0.174 |
| metformin | 27(32.1) | 28(34.1) | 30(36.6) | 0.833 |
| insulin | 20(23.8) | 16(19.5) | 19(23.2) | 0.773 |

Values are given as mean ± standard deviation (SD), median (25th–75th percentile) or number (percentage).

Tertiles of MPO levels (T1≤20.500ng/mL; 20.500<T2≤38.317 ng/ mL; T3>38.317 ng/mL)

Abbreviation: ACE, angiotensin converting enzyme; ARB, angiotensin receptor blocker; BUN, blood urea nitrogen; CRP, C-reactive protein; eGFR, estimated glomerular filtration rate; HbA1c, glycosylated hemoglobin; HDL, high-density lipoprotein; LDL, low-density lipoprotein.
