# Supplementary material for: Serum MPO levels and activities are associated with angiographic coronary atherosclerotic plaque progression in type 2 diabetic patients
Source: BMC Cardiovasc Disord. 2022 Nov 20;22:496. doi: 10.1186/s12872-022-02953-7 (PMC9677674; doi:10.1186/s12872-022-02953-7)
Supplement: Supplementary file 2 — Additional file 2. Table S2. Baseline characterize of patients with type 2 diabetes in different tertiles of MPO activity. [file 12872_2022_2953_MOESM2_ESM.docx]

**Table S2. Baseline characterize of patients with type 2 diabetes in different tertiles of MPO activity.**

|  | Diabetic patients | | | |
| --- | --- | --- | --- | --- |
| Tertiles of MPO activity | T1(n=83) | T2(n=82) | T3(n=83) | P value |
| male, n (%) | 54(65.1) | 61(74.4) | 57(68.7) | 0.424 |
| age, years | 67.33±9.20 | 65.26±8.43 | 66.27±10.16 | 0.361 |
| body mass index, kg/m^2^ | 24.61±3.12 | 25.08±3.54 | 25.39±2.89 | 0.286 |
| smoking, n (%) | 21(25.3) | 30(36.6) | 31(37.3) | 0.182 |
| hypertension, n (%) | 58(69.9) | 60(73.2) | 64(77.1) | 0.573 |
| dyslipidemia, (%) | 15(18.1) | 13(15.9) | 20(24.1) | 0.382 |
| systolic blood pressure, mm Hg | 139.37±19.57 | 138.06±21.11 | 138.98±19.28 | 0.911 |
| diastolic blood pressure, mm Hg | 74.78±11.90 | 75.65±16.24 | 75.30±10.23 | 0.912 |
| fasting blood glucose, mmol/L | 6.78±2.05 | 6.82±2.10 | 7.39±2.43 | 0.138 |
| HbA1c, % | 7.35±1.30 | 7.28±1.38 | 7.49±1.41 | 0.582 |
| serum creatinine, μmol/L | 81.20±21.08 | 76.28±18.94 | 86.94±54.80 | 0.160 |
| serum BUN, mmol/L | 7.04±3.46 | 6.47±3.20 | 6.80±3.43 | 0.553 |
| serum uric acid, μmol/L | 339.86±82.75 | 345.90±101.36 | 333.71±90.22 | 0.695 |
| eGFR, mL/min/1.73m^2^ | 79.65±17.58 | 85.61±14.69 | 80.16±19.99 | 0.056 |
| triglyceride, mmol/L | 1.68±1.15 | 1.60±0.93 | 1.61±0.83 | 0.831 |
| total cholesterol, mmol/L | 3.93±1.27 | 3.91±1.14 | 3.90±1.13 | 0.981 |
| HDL cholesterol, mmol/L | 1.08±0.30 | 1.07±0.24 | 1.05±0.28 | 0.731 |
| LDL cholesterol, mmol/L | 2.34±0.97 | 2.30±0.96 | 2.32±0.87 | 0.958 |
| apolipoprotein A, g/L | 1.22±0.20 | 1.25±0.24 | 1.19±0.24 | 0.318 |
| apolipoprotein B, g/L | 0.77±0.24 | 0.76±0.24 | 0.78±0.22 | 0.870 |
| lipoprotein (a), g/L | 0.23±0.20 | 0.24±0.28 | 0.25±0.27 | 0.865 |
| CRP, mg/mL | 0.99(0.37-3.30) | 0.86(0.35-2.23) | 1.18(0.48-3.78) | 0.276 |
| medication, n (%) |  |  |  |  |
| ACE inhibitors/ARBs | 55(66.3) | 54(65.9) | 58(69.9) | 0.831 |
| β-blockers | 41(49.4) | 49(59.8) | 49(59.0) | 0.325 |
| statins | 73(88.0) | 74(90.2) | 79(95.2) | 0.246 |
| antiplatelet | 76(91.6) | 79(96.3) | 81(97.6) | 0.162 |
| metformin | 25(30.1) | 29(35.4) | 31(37.3) | 0.598 |
| insulin | 13(15.7) | 17(20.7) | 25(30.1) | 0.075 |

Values are given as mean ± standard deviation (SD), median (25th–75th percentile) or number (percentage).

tertiles of MPO activity: T1≤6.0307mU/mL; 6.0307<T2≤9.8684 mU/ mL; T3>9.8684 mU /mL

Abbreviation: ACE, angiotensin converting enzyme; ARB, angiotensin receptor blocker; BUN, blood urea nitrogen; CRP, C-reactive protein; eGFR, estimated glomerular filtration rate; HbA1c, glycosylated hemoglobin; HDL, high-density lipoprotein; LDL, low-density lipoprotein.
