# Supplementary material for: Serum MPO levels and activities are associated with angiographic coronary atherosclerotic plaque progression in type 2 diabetic patients
Source: BMC Cardiovasc Disord. 2022 Nov 20;22:496. doi: 10.1186/s12872-022-02953-7 (PMC9677674; doi:10.1186/s12872-022-02953-7)
Supplement: Supplementary file 3 — Additional file 3. Table S3. MPO levels and activities for 1-vessel versus 2-vessel versus 3-vessel disease in patients with CHD. [file 12872_2022_2953_MOESM3_ESM.docx]

**Table S3. MPO levels and activities for 1-vessel versus 2-vessel versus 3-vessel disease in patients with CHD.**

|  | severity of CHD | | | |
| --- | --- | --- | --- | --- |
|  | 1-vessel (n=63) | 2-vessel (n=81) | 3-vessel (n=104) | P value |
| MPO level, ng/mL | 23.60(19.20-36.90) | 25.70(19.20-46.20) | 28.75(19.50-57.40) | 0.078 |
| MPO activity, mU/mL | 7.66±5.46 | 8.39±6.00 | 10.69±7.80 | 0.009 |

Values are given as mean ± standard deviation (SD) or median (25th–75th percentile).

Abbreviation: CHD, coronary atherosclerotic heart disease
